# Kinetics of arsenite removal by halobacteria from a highland Andean Chilean Salar

**DOI:** 10.1186/2046-9063-9-8

**Published:** 2013-04-01

**Authors:** Díaz-Palma Paula, Alfaro Gleny, Hengst Martha, Pozo Patricia, Stegen Susana, Queirolo Fabrizio, Rojo Gonzalo, Silva Pedro, Arias Diana, Gallardo Karem, Contreras-Ortega Carlos

**Affiliations:** 1 Departamento de Química, Facultad de Ciencias, Universidad Católica del Norte, P.O.1280, Antofagasta, Chile; 2 Advanced Mining Technology Center, AMTC, Universidad de Chile, Tupper 2007, Santiago, Chile; 3 Laboratorio de Complejidad Microbiana y Ecología Funcional, Facultad de Recursos del Mar & Centro de BioInnovación de Antofagasta, Universidad de Antofagasta, Antofagasta, Chile

**Keywords:** Arsenic, Removal, Halobacteria, Arsenite-Oxidase

## Abstract

**Background:**

The purpose of this study was to identify arsenite-oxidizing halobacteria in samples obtained from Salar de Punta Negra, II Region of Chile. Seven bacterial isolates, numbered as isolates I to VII, grown in a culture medium with 100 ppm as NaAsO_2_ (As (III)) were tested. Bacterial growth kinetics and the percent of arsenite removal (PAR) were performed simultaneously with the detection of an arsenite oxidase enzyme through Dot Blot analysis.

**Results:**

An arsenite oxidase enzyme was detected in all isolates, expressed constitutively after 10 generations grown in the absence of As (III). Bacterial growth kinetics and corresponding PAR values showed significant fluctuations over time. PARs close to 100% were shown by isolates V, VI, and VII, at different times of the bacterial growth phase; while isolate II showed PAR values around 40%, remaining constant over time.

**Conclusion:**

Halobacteria from Salar de Punta Negra showed promising properties as arsenite removers under control conditions, incubation time being a critical parameter.

## Background

Arsenic is an element distributed throughout the earth's crust, with an average concentration of 2 mg/Kg [1,2]. It is present as trace quantities in rocks, air, soil, and water [3]. This element can be found as a soluble species in nature due to natural weathering (mobilization) from rocks and soils, appearing in the form of oxyanions. This occurs mainly in two oxidation states, 3+ (arsenite) and 5+ (arsenate), and less frequently as 0 (elemental arsenic) and 3- (arsine). As^3+^ and As^5+^ named as As III and As V from now on, respectively, are mobile in the environment, the first state being more labile and toxic for most life forms [2].

In Chile, arsenic is found in most ecosystems of the northern and central area. Geology, volcanic activity, and mining operations are responsible for the presence of arsenic concentrations in surface waters and groundwater sources. The most extensive arsenic enrichment is located between 17° 30' - 26° 05' south latitude and between 67° 00' east longitude in the Andean Highlands (over 2,000 m.a.s.l.). The Atacama Desert is located in this vast region, where concentrations of total arsenic have been reported as high as 200 ppm (unpublished report).

Different water treatment technologies have been developed for arsenic removal. They are based on diverse processes such as coagulation, filtration, adsorption, and reverse-osmosis [4]. However, most of these technologies target As (V), thus requiring a pre-oxidation step to change As (III) into As (V). This step is usually performed by employing powerful chemical oxidants (ozone, chlorine, or H_2_O_2_), which have different toxicity degrees [5]. Thus, the bacterial biotransformation of toxic arsenic compounds becomes a more friendly alternative for human and environmental health than chemical remediation.

The importance of microorganisms in arsenic transformation and its biogeochemical cycle has recently received much attention [6-12]. Most of the microorganisms involved in As transformation cycling live in places where, naturally or anthropogenically, the concentrations of this metalloid in its most toxic form (As (III)) are high. Therefore, these microorganisms should be able to change the arsenic oxidation states by chemically transforming it in order to survive. The capability of bacterial species to transform arsenite to arsenate is due to the presence of the arsenite oxidase enzyme (AOX), which was extracted, purified, and characterized for the first time from *Alcaligenes faecalis* strains [6,7]. Subsequently, arsenite oxidase has been found in other bacterial strains and studied in detail (eg. *Pseudomonas alcaligenes*, *Agrobacterium tumefaciens*, *Alkalilimnicola ehrlichii, Wautersia solanacearum*, *Acinetobacter calcoaceticus*, and *Enterobacter cloacae*). Some bacteria gain energy from arsenite oxidation [8-10,12]; however, this activity would be limited, on exceptional grounds, to chemolithoautotrophic bacteria. On the other hand, energy gained through heterotrophic bacteria experimentally grown with arsenite has yet to be proven [10,11]. Though there is a great diversity of arsenite-oxidizer bacteria well handled under laboratory conditions, experimental evidence shows that it is dificult for these bacterial strains to adapt to field conditions, where arsenite removal is needed. In these cases the optimal strategy is the use of *in-situ* bioremediation, which involves growth stimulation of native bacterial flora having metabolic capabilities to biotransform arsenite. Therefore, it is desirable to search for autochthonous organisms leading to *in-situ* bioremediation strategies, by making site-specific studies.

A high functional diversity of extremophiles microorganisms is developed in Highland Andean ecosystems [13]. These microbial communities are adapted to high arsenic charges present in a natural form in water and in sediments, therefore it are a source of researching looking for bio-transforming the toxic arsenic species. Both spring waters and shallow hypersaline lagoons from Salar de Punta Negra, located in the Andean Highlands of Chile, are naturally enriched with arsenic, which reaches above 100 ppm/L. To investigate the presence of arsenite-oxidizing halobacteria in samples obtained from this place and eventually the existence of enzyme arsenite oxidase in these bacteria, their capability to remove As (III) along with the kinetics of bacterial growth for its potential use in *in-situ* bioremediation strategies were evaluated. The presence of the enzyme arsenite oxidase was analyzed by using molecular techniques.

## Results

**Figure 1:**
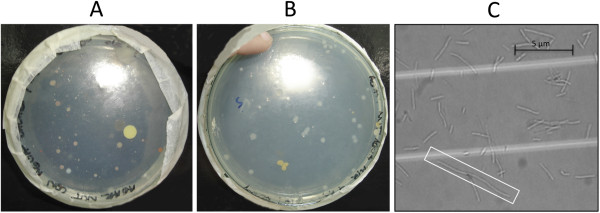
**Morphological Characterization of Bacterial Isolates from Salar de Punta Negra. A**: Colonies from G_1_ in IM Media; **B**: Colonies from G_1_ in CIM Media, and **C**: Bacteroid, or non- segmented elongated bacterial cell (rectangle) present in GM/S Media.

Characterization of Bacteria Isolates: After 18 h of culture, different colonies grew on IM and CIM media plates. There were no significant differences in number and/or shape among colonies grown in either culture media (Figures 1A and 1B). Thirteen morphotypes from all the colonies were selected according to their aspect (appearance, color, shape, average diameter, and edge characteristics). They were characterized by Gram stain. All isolates corresponded to strepto-bacillus gram-negative bacteria. Additionally, microscopic analyses revealed the presence of bacteroides or long-sized non-segmented bacteria (Figure 1C).

Based on the digestion with restriction enzymes of 16S rRNA gene, the 13 isolates determined by RFLP analysis actually matched with 7 different isolates. RFLP results were used as identification criteria for these 7 isolates, by using Roman numerals from I to VII.

**Figure 2:**
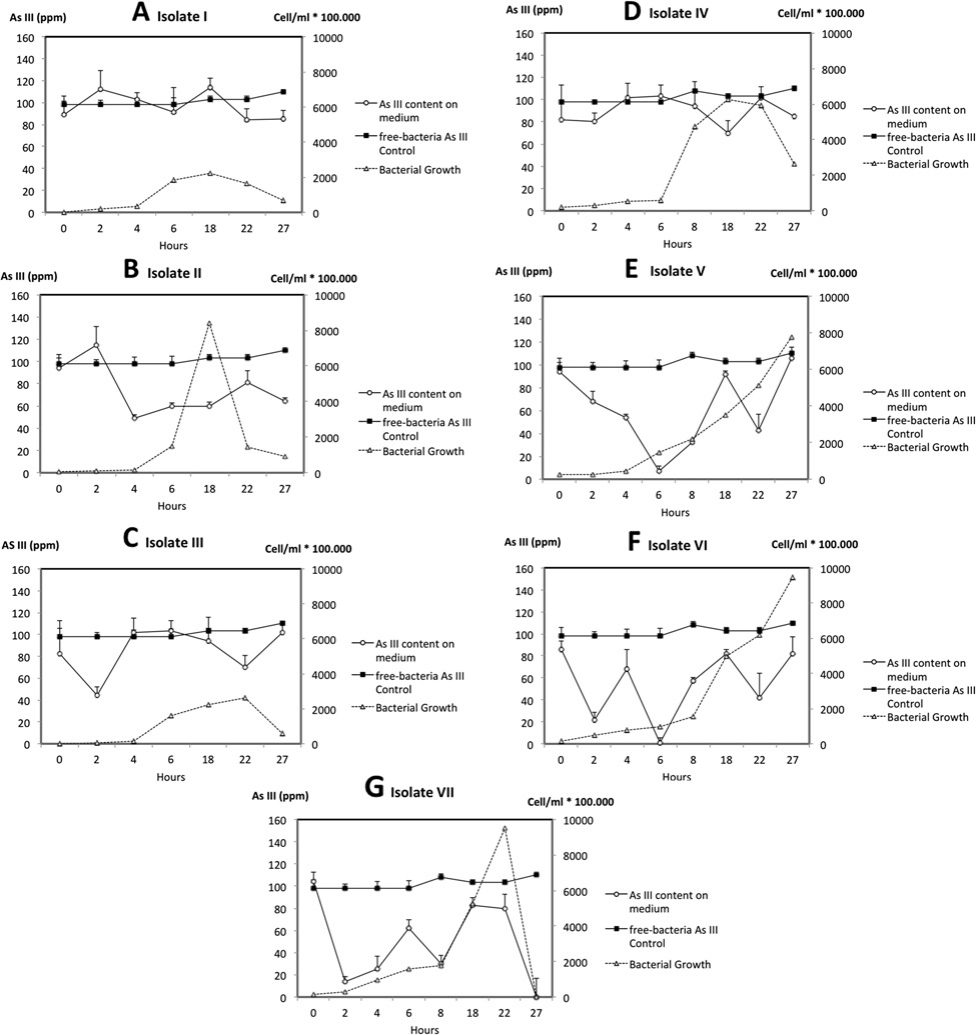
**Arsenic and growth kinetics of isolates from Salar de Punta Negra from G**_**13**_**, grown on GM/S during 27 h. A**: Isolate I; **B**: Isolate II; **C**: Isolate III; **D**: Isolate IV; **E**: Isolate V; **F**: Isolate VI, and **G**: Isolate VII.

**Figure 3:**
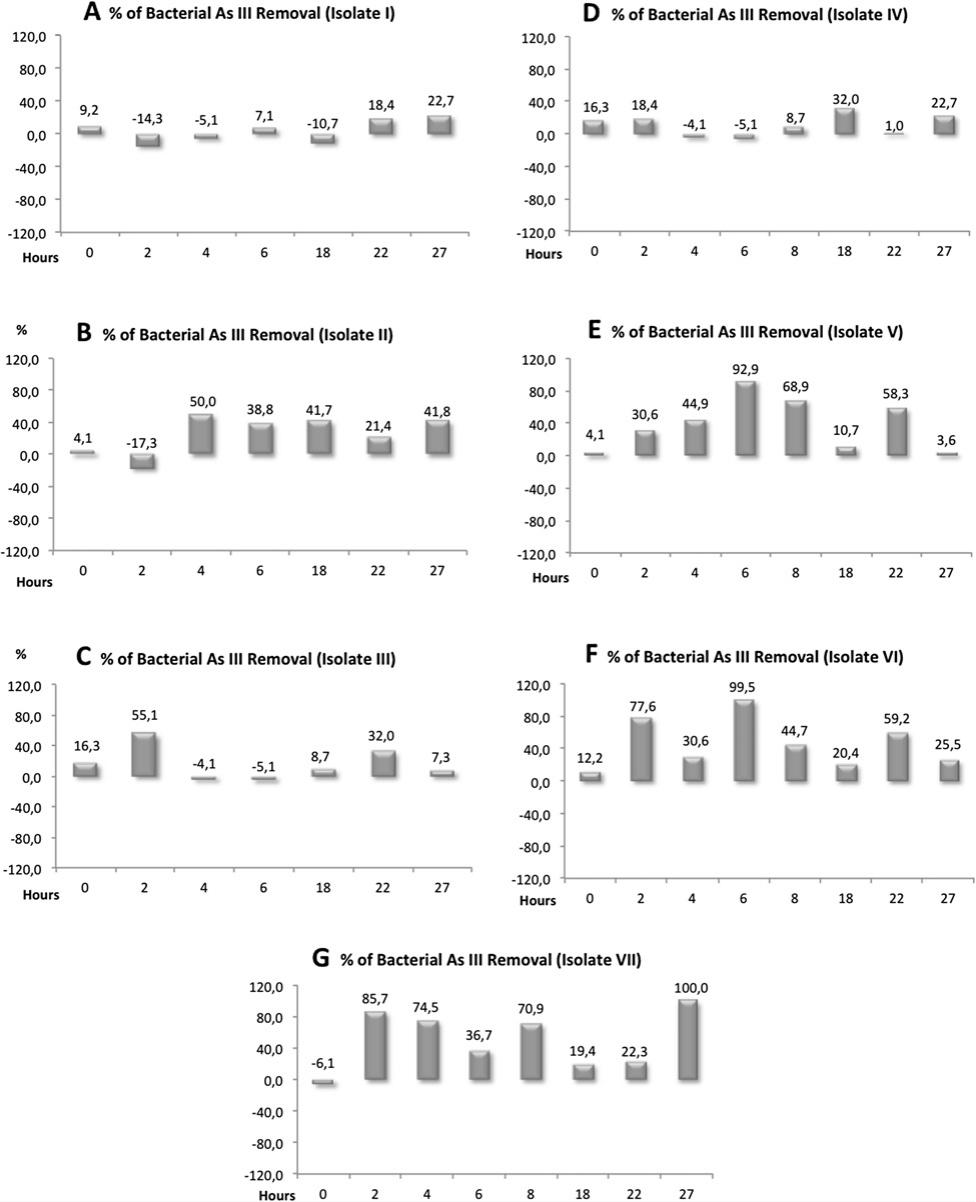
**Percent of Arsenite Removal (PAR) by isolates from Salar de Punta Negra from G**_**13**_**, grown on GM/S during 27 h. A**: Isolate I; **B**: Isolate II; **C**: Isolate III; **D**: Isolate IV; **E**: Isolate V; **F**: Isolate VI, and **G**: Isolate VII.

Arsenic and Bacterial Growth Kinetics: Figure 2 shows the fluctuation of As (III) concentration values measured in treatments (isolates I to VII) and controls, together with bacterial growth curves for each isolate. Data at t_8_ are not shown in figures and tables containing arsenite values (Figures 2, 3 and Table 1) because variability among replicates was too large to be considered.

**Table 1:** Discrete values of as (iii) for kinetic analysis and validation results for as (iii) method

**As (III) values for treatments and controls (Average for 3 replicates +/- Standard Desviation)**								
**t**_**n **_**(hours)**	**t**_**0**_	**t**_**2**_	**t**_**4**_	**t**_**6**_	**t**_**8**_	**t**_**18**_	**t**_**22**_	**t**_**27**_
Isolate I	89 ± 12.0	112 ± 17.1	103 ± 6.0	91 ± 23.0	NRD	114 ± 8.0	84 ± 10.0	85 ± 8.1
Isolate II	94 ± 9.0	115 ± 16.2	49 ± 2.8	60 ± 2.4	NRD	60 ± 3.6	81 ± 11.1	64 ± 3.0
Isolate III	82 ± 31	44 ± 7.9	102 ± 13.0	103 ± 10.0	NRD	94 ± 22.0	70 ± 11.0	102 ± 9.6
Isolate IV	82 ± 6.7	80 ± 12.0	102 ± 8.3	103 ± 8.0	94 ± 7.6	70 ± 11.5	102 ± 16.4	85 ± 8.1
Isolate V	94 ± 8.3	68 ± 8.7	54 ± 2.5	7 ± 4.3	32 ± 1.1	92 ± 2.6	43 ± 14.0	106 ± 9.8
Isolate VI	86 ± 7.8	22 ± 6.3	68 ± 18.0	0.52 ± 5.1	57 ± 3.3	82 ± 3.9	42 ± 22.0	82 ± 15.1
Isolate VII	104 ± 8.3	14 ± 4.5	25 ± 12.1	62 ± 8.0	30 ± 7.7	83 ± 6.6	80±12.6	0.001 ± 17.1
Free-bacteria Control	98±8.0	98 ± 4.0	98 ± 6.0	98 ± 6.7	108 ± 3.0	103 ± 3.0	103 ± 3.0	110 ± 0.6
***Data and Validation analysis for control replicates (free-bacteria control)***								
Average	102							
Standard Deviation	5.0							
r limit	12.75460701							
R limit	14.28977879							
Detection limit	0.001							

All values are expressed in ppm; *NRD*: Non-reliable data were obtained for this incubation time.

Arsenite values presented great variation during the experimental time (t_0_ –t_27_), showing arsenite reductions for all isolates as compared to the control, at the same incubation time (Figure 2, A to E). Isolates VI and VII showed reductions at all incubation times (t_0_ –t_27_); isolate V showed reductions up to t_27;_ and isolate II from t_4_ to t_27_. Some of these measurements were close to the detection limit of the method (0.001 ppm), showing values close to zero, revealed at 6 h (t_6_) for isolate VI and at 27 h (t_27_) for isolate VII (Table 1). However, these so deep slumps in As (III) levels were not recorded in the rest of the experiment (Figure 2). Exceptionally, isolate II exhibited arsenite reduction whose magnitude remained almost constant from t_4_ to t_27_ (Figure 2B). Minor decreases of arsenite levels were recorded for isolates I, II, III and IV (Figure 2, A to D).

Though no clear correspondence was found between bacterial growth and arsenite decrements in the culture medium, for clones showing the largest arsenite reductions, these occurred during lag phase of growth (Figure 2, E, and F in the interval t_0_-t_6_ and Figure 2G in the interval t_0_-t_2_). It is also observed that for isolates II, IV, V, VI, and VII, bacterial growth and arsenite variation follow parallel trends between t_22_ and t_27_ (Figure 2, B, D, E, F, and G); while for isolates I and III, trends follow opposite directions in the same time interval (Figure 2, A and C) as well as for clone IV between t_6_-t_8_.

Data did not show significant arsenite variations in the free-bacterial control over the whole analysis period, showing an arsenite concentration average of 102 ± 5 ppm (Table 1). On the other hand, validation criteria applied to the arsenite measurements of free-bacteria controls indicated that data are repeatable and reproducible as they comply with the limits of Repeatability (r) and Reproducibility (R) between series. Thus, results do not show a difference greater than 12.754 ppb and 14.289 ppb, respectively (Table 1).

Percentage of Arsenite Removal: Figure 3 (A to G) shows the arsenite kinetics presented as Percentage of Arsenite Removed (PAR) from the culture media versus incubation time. The range of variation for PARs fluctuated between negative values to approximately 100%. These negative PAR values of PARs estimated for isolates I, II, III, IV, and VI correspond to a calculation artefact based on the dispersion arsenite values themselves, which in some cases showed higher mean values at some t_n_ than at t_0_. For example, isolate I had a higher value in t_0_ than at t_2_ (Table 1), but the former showed a high standard deviation, so that its value could also be equal to or less than t_0_.

On an average, the highest values of PARs were detected in the cultures of isolates V, VI, and VII. However, it is noteworthy that there was a high variability over time (Figure 3). Notoriously, isolate V showed a steady increase in PAR values between t_0_ and t_6_ (Figure 3). Furthermore, although isolate II showed low PARs, values remained relatively stable between t_4_ and t_27_ (Figure 3). Isolate VII presented a maximum PAR value of 100% at t_27_. Though PAR values should be zero at t_0,_ they fluctuate between -6 and +16. So, all PAR values at Figure 3 should be subjected to such variation. In fact, PAR values show standard deviations around 12%.

**Figure 4:**
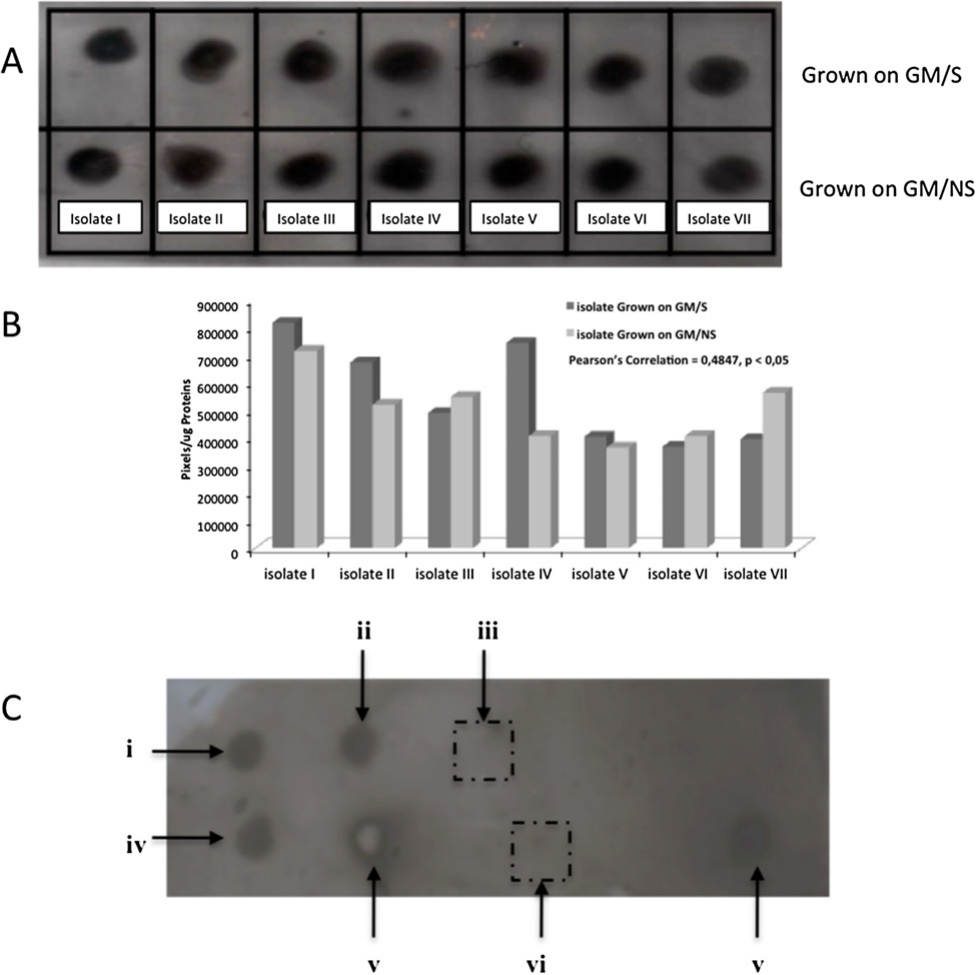
**Dot blot analysis for G**_**13 **_**clones from Salar de Punta Negra. A**: isolates grown with and without As (III); **B**: comparisons of experimental dot pixels from image analysis as a function of protein concentration, and **C**: Dot Blot controls (i): isolate II grown with arsenite; (ii): isolate II grown without arsenite; (iii): Dot Medium control; (iv): Dot *Agrobacterium tumefaciens* C58/ATCC 33970; (v): Dot arsenite oxidase purified from *Alcaligenes faecalis* NCIB 8687 and (vi): Dot xanthine oxidase.

Arsenite Oxidase and Protein Analysis: Dot Blot performed with lysates of G_13_–bacterial treatments, cultured with and without NaAsO_2_ (Figure 4A), revealed that there was antibody recognition for arsenite oxidase in all cases. This means that the expression of the enzyme in the halobacterium is not necessarily induced by the presence of arsenite in the growth medium. Figure 4B shows a similar dot-pixel analysis as a function of protein concentration for the different isolate treatments for, i.e., with and without arsenite. An exception is made for isolate IV which showed a higher expression when grew in GM/S and a low expression in GM/NS (Figure 4B). This means that arsenite seems not to influence the arsenite oxidase expression relative to total protein concentration (pixels/μg proteins). Figure 4B also shows that arsenite oxidase concentrations are the lowest for isolates V, VI, and VII, showing the highest average arsenite removals. It is worth noting that those values were taken at t_18_, when the As III values were high for this isolates. In relation to controls, a positive antibody recognition with lysates of *A. tumefaciens* C58/ATCC 33970 (Figure 4C, v) and with arsenite-oxidase purified from *Alcaligenes faecalis* NCIB 8687 (Figure 4C, iv) was obtained, whereas there was no recognition with control medium (iii). Moreover, Figure 4 shows that there was no cross-reaction with purified enzyme xanthine oxidase (vi), the result thus revealing the specific presence of arsenite oxidase in the lysates from Salar de Punta Negra.

## Discussion

Isolation and Bacterial Growth: The similar number and type of colonies grown on isolation media, both with and without arsenite (IM and CIM, respectively), seem to indicate an adequate adaptation of isolated bacterial populations to a medium containing arsenite, despite the high toxicity induced in the medium. This adaptation could be facilitated by the fact that the isolation media was prepared with steril water from Salar de Punta Negra in order to keep the complex salt balance of the collection site [14]. Thus, the ionic balance of Andean saline lakes is an additional restriction for attaining biodiversity in laboratory cultures since this aspect is essential for the development of halophylic microbial communities, such as Salar de Punta Negra, an issue that remains poorly studied.

Considering bacterial adaptation to As III, the presence of elongated bacteroids could indicate a cellular response to metabolic stress generated by exposure to arsenic. This result is consistent with those described by different authors [15-19], relative to heavy metals inhibiting the cleavage of the bacterial cell wall, without discontinuing other processes such as growth of the same wall, growth of the cell membrane, and synthesis of individual cellular components.

Arsenic and Bacterial Growth Kinetics: Clearly, bacteria isolated from Salar de Punta Negra were able to grow with high NaAsO_2_ values. Furthermore, bacteria growth was associated with a decrease of As (III) in the medium in some cases, even though this reduction did not remain over time, as revealed by the bacterial growth kinetics examined in this study. In addition, no reductions of arsenite levels in free-bacterial controls indicate that bacteria produced real decrements of this element in the media. It is important to highlight that the neutral pH conditions of the media (Table 2) could not promote chemical transformation between different arsenic oxidation states, despite the spontaneous transformation of As (III) into As (V) under aerobic conditions predicted in the theory [20,21].

**Table 2:** Culture media composition

**Isolation Media**	**Control Isolation Media**	**Growth Media suplemented with As**	**Growth Media non-suplemented with As**	**Luria Media**
**(IM)**	**(CIM)**	**(GM/S)**	**(GM/NS)**	**(LM)**
- Nutritive Agar	- Nutritive Agar	-Peptone Broth	-Peptone Broth	- Tryptone
(DIFCO).	(DIFCO).	(DIFCO).	(DIFCO).	-Yeast extract
- Collection site water filtered through 0.2 um	- Collection site water filtered through 0.2 um	-Sterilized Milli-Q Water	-Sterilized Milli-Q Water	-NaCl 0,1M
- NaAsO_2_ 100 ppm	- pH = 7,0	- NaAsO_2_ 100 ppm	-pH = 7,0	- NaOH 2M to adjustment pH at 7,0
- pH = 7,0		- pH = 7,0		- Sterilized Milli-Q Water
				- NaAsO_2_ 100 ppm.

The variations observed in the arsenite levels of bacterial cultures could indicate the existence of oxidation-reduction and biosorption-biodesorption mechanisms of arsenic occurring simultaneously, as described by Silver and Phung (2005) [22]. Here, the authors refer to reports describing a broad diversity of microbes able either to reduce arsenate or to oxidize arsenite, through co-functioning of the arsenite oxidase and arsenate reductase membranous enzymatic systems [23-26]. Although there is no consensus on which of the variables triggers the prevalence of one mechanism over the other, some of the variables described as key to explain the changes in arsenic metabolism would be pH [27], oxygen concentrations [25], and carbon sources and temperature, among others [27], inducing the expression of genes associated with arsenic metabolism [28]. Even though the coexistence of arsenite-oxidizing and arsenate-reducing bacteria [29] would be considered as a difficulty to implement biorremediation, specific protocols to stimulate the predominance of oxidizing processes, like the maintenance of aerobic and neutral pH conditions in the environment surrounding the bacteria, suggest ways to achieve stable arsenite removal via microbial arsenite oxidation over time.

Arsenite Oxidase Detection Essays: Dot blot analysis showed the presence of arsenite oxidase in both arsenic-baited bacteria and arsenic non-baited bacteria. Indeed, kinetic essays showed arsenite reductions starting early, during the lag phase of bacterial growth, which is an indicator that arsenite oxidase is constitutively expressed by Salar de Punta Negra bacterial isolates. This was observed in 5 out of 7 cases (See Figure 2). There are few cases reported for this condition since, in most cases, arsenite is required in the growth medium for arsenite oxidase gene expression [30,31]. *Thiomonas* sp. str. 3As; *Agrobacterium tumefaciens* str. 5As and the microbial biofilm GM1 (isolated from arsenite-containing acid mined drainage waters of Giant Mine in Canada) also express the arsenite oxidase when the organisms are growing in the absence of arsenite, but in all of these cases, the expression does not occur before the logarithmic phase [32-34]. As mentioned above, for most clones in this study the arsenite oxidase activity was detected in the lag phase, removing As (III) medium up to 99%. The explanation for expressing the enzyme earlier by isolates can be as complex as the genetic regulatory mechanisms recently described for *A. tumefaciens* strains involving As (III) sensing, components of signal transduction, and quorum sensing [33].

## Conclusions

This paper gives evidence of arsenite oxidation by bacteria present in a High-Andean hypersaline ecosystem (arsenite-oxidizing strains have also been reported in the coastal territory in northern Chile [12]). The studied sampling site, Salar de Punta Negra, is an extreme environment regarding arsenic concentrations, which reach values as high as 10 mM in current and underground waters [14]. Arsenite oxidizers were isolated from hypersaline sediments and a group of halofilic bacteria with different arsenite bioremediation potential was found. Thus, 3 arsenite-oxidizing isolates (V, VI, and VII) were able to grow, removing nearly 100% initial arsenite in the medium, but displaying a high variability of this capability over the culture time. Another isolate (II) was capable of removing about 40% inicial arsenite during its logarithmic phase, exhibiting a notable stability of its capacity over time. In all these cases the arsenite oxidase was constitutively expressed.

Studies show that halobacteria from Salar de Punta Negra exhibit promising properties as arsenite removers under control conditions, where incubation time is a critical parameter and, particularly, in those situations where *in-situ* arsenite removal is needed.

## Methods

Description of Sampling Site: Salar de Punta Negra is a 250 Km^2^ athalassohaline wetland located in the Atacama Desert, northern Chile (24^o^ 28' S, 69^o^ 53' W, at 2,976 m.a.s.l.). It consists of several temporary saline lagoons covering about 3 Km^2^. Mining activities occurring around this place obtain most of the water for their processes from the groundwater below the salt flat through a network of extraction wells. The geographic and climatic characteristics of the Salar together with their chemical composition provide ideal conditions for the growth of extreme microorganisms, such as bacteria, archaea, and microalgae [35], which are the trophic bases of a unique aquatic flora and fauna (microalgae, microcrustaceae, rotifers, and flamingos). Water and sediment samples from 2 lagoons located in the eastern border of the Salar were collected using sterile spatulas and sterile plastic receptacles. Samples were transported in cooler boxers and processed immediately at the Biochemistry Laboratory of Universidad Católica del Norte.

Growth Media and Bacterial Isolation: Samples from the Salar were incubated in different media with and without sodium meta-arsenite (NaAsO_2_) in order to isolate and grow microorganisms. These media and their composition were named as Isolation Media (IM), Control Isolation Media (CIM), Growth Media Supplemented with As III (GM/S), and Growth Media Non Supplemented with As III (GM/NS), respectively, as shown in Table 2. A strain of *Agrobacterium tumefaciens* C58/ATCC 33970 was cultured in Luria broth and employed as positive control for arsenite oxidase detection. It was named Luria Media (LM). In order to promote the adaptation of isolated microorganisms and obtain a representative fraction of the microbial biodiversity in each sample, the isolation medium was prepared with sterile-filtered water from Salar de Punta Negra (SWS). This was done so as to make bacterial growth possible and maintain the chemical equilibrium present in the water from Salar de Punta Negra in these media. Thus, the nutritive agar (DIFCO) was dissolved in a minimal milli-Q water volume sterilized at 121°C/1 atm. Then, the volume was completed with SWS at 60°C. Table 2 shows the details of media composition. To obtain GM/S and GM/NS, growth media were prepared with a basis of Peptone Broth (DIFCO) supplemented and non-supplemented with NaAsO_2_^.^H_2_O (Merk), respectively. For GM/S preparation a sterile-filtered NaAsO_2_^.^H_2_O solution was added at 60°C, after autoclaving the media. The resulting concentration in the GM/S was 100 ppm in As III, prepared from a stock solution of NaAsO_2_^.^H_2_O 200 ppm.

Given the pKa of As(OH)_3_ (9.3) and the neutral pH of culture media, this compound dissociates according to equation 1, with an oxidation state 3+:

(1) $${\mathrm{NaAsO}}_{2}+H_{2}O\to {\mathrm{Na}}^{+}{}_{\left(\mathrm{aq}\right)}+H_{2}{\mathrm{AsO}}_{3}{{}^{-}}_{\left(\mathrm{aq}\right)}$$

A mixture of surface sediments and water from Salar de Punta Negra was diluted in SWS in a 1:1000 ratio. This diluted mixture was named as dilute sample. Ten plates containing IM were inoculated with 1 mL of the previous dilute sample and incubated at 30°C until the presence of colonies, named here as “generation zero” (G_0_), was verified. Then, colonies from this generation, with different morphology (color, edge, and height), were transferred to plates with GM/S and incubated at 30°C for 18 to 24 h. The colonies obtained in this step were named as G_1_ and compared with the colonies of G_0_, through a Gram test. To increase the biomass required for the next experiments, colonies from G_1_ were transferred to flasks with 200 mL of GM/S until obtaining a new bacteria generation(G_2_).

Molecular Identification of Isolates: In order to determine the genomic variability between bacterial colonies, a restriction fragment length polymorphism analysis (RFLP) was conducted. Genomic DNA from isolates were obtained by Power Soil DNA isolation Kit (MoBio Laboratories Inc., Solana Beach, CA, US) and amplified by PCR. 16S rRNA genes were amplified using the primer pairs specific for Bacteria 8F (5^′^ AGAGTTTGATCCTGGCTCAG 3^′^) and 1392R (5^′^ ACGGGCGGTGTGTAC 3^′^), using 10 ng of DNA as a template in each amplification reaction. PCR conditions were selected as previously described by Norton et al. (2008) [35]. About ~1,500 bp amplicons were digested with *HhaI* and *MspI* restriction enzymes in separated reactions for 3 h at 37°C. Restriction patterns were compared by electrophoresis in 2% agarose gel and stained with etidium bromide.

Generational Cultures: To determine both, the presence of the arsenite-oxidase enzyme in bacterial lysates and if it was inducible in the presence of As (III), clones selected by RFLP analysis were cultured for several generations in media with and without NaAsO_2_. Thus, bacterial pellets of each of the seven isolates selected from G_2_ (10 μL) were inoculated in 200 mL of GM/S and GM/NS media (Table 2) and incubated for 18 h at 30°C to increase the biomass (G_3_)_._ This procedure was repeated 10 times for each isolate to reach generation number 13^th^ or G_13_. At the same time, the reference strain of *Agrobacterium tumefaciens* C58/ATCC 33970 was grown in LM, according to the protocol described above (Table 2). This bacterium was used for the Dot Blot experiments as a positive control for the expression of arsenite oxidase [36]. Finally, Bradford Analysis [37] was performed for total protein quantification from each isolate of G_13_.

Arsenic Kinetic Experiments: In order to determine the correlation between bacterial growth and As (III) removal from the culture media, the kinetics of 7 isolates from G_13_ was performed in separate experiments. Thus, 10 μL of pellets from each bacterial isolate were inoculated in screw cap tubes with 15 mL of GM/S and incubated at 30°C for 18 h. Once bacterial growth was obtained, 100 μL of bacterial inoculation were transferred to Erlenmeyer flasks containing 200 mL of GM/S and incubated at 30°C, while stirring at 300 rpm for 36 h. Medium controls consisting of 200 mL non-inoculated GM/S were incubated together with the previous experimental treatment flasks under the experimental conditions above to monitor the concentration of As (III) in the supernatants of the treatment flasks, relative to the total As. The latter is represented by arsenite concentration in the control medium since it does not suffer any significant change during the experiments.

As (III) in the culture medium of each treatment flask and the medium control were measured after bacteria removal, at specific incubation times: t_0_, t_2_, t_4_, t_6_, t_8_, t_18_, t_22_, t_27_, t_30,_ and t_36_. To do this, 10 mL from each culture flask were filtered through sterile disposable filters of 0.22 μm pore. The filtered-recovered medium was used for arsenic measurements. The same procedure was used for the control culture flasks. Both, treatment and control flasks, were analyzed in triplicate. Simultaneously with arsenic measurements, non-filtered samples from the treatment flasks were used to estimate bacterial density. For this purpose, the samples were fixed in buffered glutaraldehyde (2% glutaraldehyde solution in 0.05 M phosphate buffer at pH 7.2-7.4) and cell counts were determined by the Neubauer chambers technique.

The arsenite removal capabilities of bacteria were expressed as Percentage of Arsenite Removal (PAR) and calculated from the initial values of As (III) present in the culture media (t_0_) and their corresponding values at each incubation time.

Arsenite/Total-Arsenic Quantification: Total-As and As (III) measurements were carried out by hydride-generation flow-injection atomic absorption spectrophotometry (FIAS-HG-AAS) with a detection limit of 0.001 ppm. This is a suitable method for the analysis of arsenic in waters, and it is used in most laboratories because of its high sensitivity, speed of analysis, and comparatively low cost [38,39]. A basic validation analysis was applied to the data by calculating Reproducibility and Repeatability limits [40].

Arsenite Oxidase Dot Blot Analysis: A dot blot analysis was conducted for arsenite oxidase detection. Bacterial pellets of G_13_ at exponential growth phase were re-suspended in 4 mL of sterilized Milli-Q Water and lysed by sonication (140 Hz) with 3 30-s cycles on ice. Once the bacterial pellets were lysated, suspensions were transferred to 10 mL sterilized tubes. Then, 20 μL of 3 different protease inhibitors were added: aprotin (1 mg/mL); leupeptine (1 mg/mL); and PMSF/DMSO (100 mM). Five μL of each lysated were put on a nitrate-cellulose membrane (pore size of 0.47 mm Millipore). To check the specificity of antibodies and prevent cross reactivity, another 5 μL of bovine xantine oxidase (another molibdopterin protein) from bovine milk, Sigma – Aldrich diluted in 1:50, were dotted on the same membrane. This enzyme was chosen because it exhibits a high structural similarity with arsenite oxydase [14]. Arsenite oxydase purified from *A. faecalis* NCBI 8687 (792 Ao, 55 μM) [6], provided by Dr. Gretchen Anderson (Department of Chemistry, Indiana University, South Bend in USA), and lysates of *A. tumefaciens* C58/ATCC 33970 were used as positive controls of AOX expression. Sterilized GM/S was used as negative control for the same purpose. The membrane was blocked with 6% non-fat milk in TBS (0.1% Tween 20) (TBS-T) for 2 h at room temperature. To detect the arsenite oxidase, the membrane was incubated overnight at 4°C with diluted 1:25 in PBS-T polyclonal rabbit anti *A. faecalis* AOX primary antibody (793 ARs, 100 μg/ml), also provided by Dr. Gretchen Anderson. Then, the membrane was washed 3 times with TBS-T and incubated with 0.2 μg/mL HRP-conjugated goat anti-rabbit IgG for 1.5 h. They it was washed again and revealed with Pierce ECL Substrate and GE Healthcare ECL Reagent prepared according to the manufacturer’s instructions.

Both microbiological and physicochemical assays of the present study were carried out following security protocols normalized in the Laboratorio de Servicios Analíticos (LSA) at the Universidad Católica del Norte wich has the ISO/IEC 17025 accreditation [41].

## Abbreviations

PAR: Percentage of arsenite removal; IM: Isolation medium; CIM: Control isolation medium; GM/S: Growth media supplemented with arsenite; GM/NS: Growth media non supplemented with arsenite; LM: Luria medium; Gn: Generation n; tn: Time n; SWS: Sterile-filtered water of salar de punta negra

## Competing interests

No competing interests are involved in this work.

## Authors’ contribution

D-PP: Conceived the study and participated in its design and coordination. She also wrote the core of the manuscript. GA; PS, and GR: They conducted the microbiological analysis and the As kinetics. They participated as undergraduate students. MH: She conducted the molecular genetic studies for clone identification, RFLP analysis and helped write the manuscript draft. PP: She coordinated the dot blot analysis and helped write the manuscript draft. SS, FQ, and CC: She coordinated the As Analysis and validation methods for As data. DA and KG: They conducted the Dot Blot analysis. All authors read and approved the final manuscript.

## Authors' information

The main author, D-PP, has always worked in environmental microbiology concerning saline environments of arid zones in Chile and Spain. In the last few years, she has been part of a research group including analytical chemists (GA, SS, CF), physical chemists (CC), and biochemists (PP and MH), who have been studying stress factors affecting the biological communities of Chilean and Peruvian Highlands (heavy metals, phytochelantins, etc.). Together with Doctors SS, FQ, and DC, she is part of the research group at UCN Analytical Services Laboratory, the only testing lab accredited by ISO 17,025 in northern Chile. In addition, Dr. FQ is the president of the Analytical Chemistry Division of the Chilean Chemical Society. All students, DA, KG, GR, and PS, who participated in this study, prepared their undergraduate and graduate theses.
